# Identification of an intronic enhancer regulating RANKL expression in osteocytic cells

**DOI:** 10.1038/s41413-023-00277-6

**Published:** 2023-08-11

**Authors:** Minglu Yan, Masayuki Tsukasaki, Ryunosuke Muro, Yutaro Ando, Kazutaka Nakamura, Noriko Komatsu, Takeshi Nitta, Tadashi Okamura, Kazuo Okamoto, Hiroshi Takayanagi

**Affiliations:** 1 Department of Immunology, Graduate School of Medicine and Faculty of Medicine, The University of Tokyo, Tokyo, Japan; 2 Department of Osteoimmunology, Graduate School of Medicine and Faculty of Medicine, The University of Tokyo, Tokyo, Japan; 3 Department of Microbiology, Tokyo Dental College, Tokyo, Japan; 4 Department of Oral and Maxillofacial Surgery, Department of Sensory and Motor System Medicine, Graduate School of Medicine, The University of Tokyo, Tokyo, Japan; 5 Department of Laboratory Animal Medicine, Research Institute, National Center for Global Health and Medicine, Tokyo, Japan

**Keywords:** Bone, Metabolism

## Abstract

The bony skeleton is continuously renewed throughout adult life by the bone remodeling process, in which old or damaged bone is removed by osteoclasts via largely unknown mechanisms. Osteocytes regulate bone remodeling by producing the osteoclast differentiation factor RANKL (encoded by the *TNFSF11* gene). However, the precise mechanisms underlying RANKL expression in osteocytes are still elusive. Here, we explored the epigenomic landscape of osteocytic cells and identified a hitherto-undescribed osteocytic cell-specific intronic enhancer in the *TNFSF11* gene locus. Bioinformatics analyses showed that transcription factors involved in cell death and senescence act on this intronic enhancer region. Single-cell transcriptomic data analysis demonstrated that cell death signaling increased RANKL expression in osteocytic cells. Genetic deletion of the intronic enhancer led to a high-bone-mass phenotype with decreased levels of RANKL in osteocytic cells and osteoclastogenesis in the adult stage, while RANKL expression was not affected in osteoblasts or lymphocytes. These data suggest that osteocytes may utilize a specialized regulatory element to facilitate osteoclast formation at the bone surface to be resorbed by linking signals from cellular senescence/death and RANKL expression.

## Introduction

Osteocytes are the most abundant bone cells, accounting for 90%–95% of all bone cells, and the longest-living cell type in bone tissue.^1^ Osteocytes regulate bone remodeling by regulating both osteoclast and osteoblast activity via expression of key factors such as RANKL and sclerostin.^2–6^ Previous studies using *Tnfsf11*-floxed mice crossed with *Dmp1*-Cre and *Sost*-Cre mice have shown that osteocyte RANKL is essential for physiological bone remodeling in the adult stage^2–4^ and contributes to pathological bone loss induced by estrogen deficiency,^7^ unloading,^3^ hyperparathyroidism^8,9^ and glucocorticoid treatment.^10–12^ However, the precise molecular mechanisms underlying RANKL expression in osteocytes remain poorly understood.

Bone remodeling begins with the removal of old or damaged bone by osteoclasts, which is followed by the formation of new bone by osteoblasts.^1,4^ Although this is one of the fundamental concepts in bone biology, the molecular signals driving osteoclastogenesis on the surface of bone that need to be renewed are still largely unknown. Due to their matrix-embedded and long-lived nature, osteocytes contribute to the sensing of mechanical loading and the detection of microdamage, thus playing a key role in the response to bone tissue senescence.^1^ Osteocyte death is associated with various conditions, such as microdamage, disuse, estrogen loss, glucocorticoid treatment, inflammatory diseases and aging.^1^ A previous study has shown that bone tissue that contains dying osteocytes displays an increased RANKL expression level.^13^ Dying osteocytes have been shown to release adenosine triphosphate (ATP) to stimulate RANKL expression in neighboring bystander osteocytes.^14^ Since phagocytes cannot reach and engulf apoptotic osteocytes embedded in the bone matrix, apoptotic osteocytes undergo secondary necrosis and release damage-associated molecular patterns (DAMPs), which can promote RANKL-induced osteoclastogenesis.^15^ The senescence-associated transcription factor (TF) GATA4 stimulates RANKL expression in osteocytes, leading to bone loss in adult mice.^16,17^ These studies suggest that dying and senescent osteocytes may produce RANKL to induce osteoclast formation at the sites at which renewal is needed. However, it remains unknown how signals from cell death and senescence are integrated into the genomic region of RANKL and thus stimulate its expression in osteocytes.

In this study, we analyzed the chromatin landscape of various RANKL-producing cell types and identified an intronic RANKL enhancer specifically activated in osteocytic cells, including osteocytes and late-stage osteoblasts. TF binding site analysis suggested that the TFs associated with cell death and senescence activate the intronic enhancer element. Mice lacking the intronic enhancer region exhibited decreased RANKL expression specifically in osteocytic cells, leading to a high-bone-mass phenotype in the adult stage. These data provide a molecular basis for RANKL regulation in osteocytic cells and may have therapeutic implications for various skeletal disorders.

## Results

### Identification of an intronic enhancer of RANKL in osteocytic cells

**Fig. 1:**
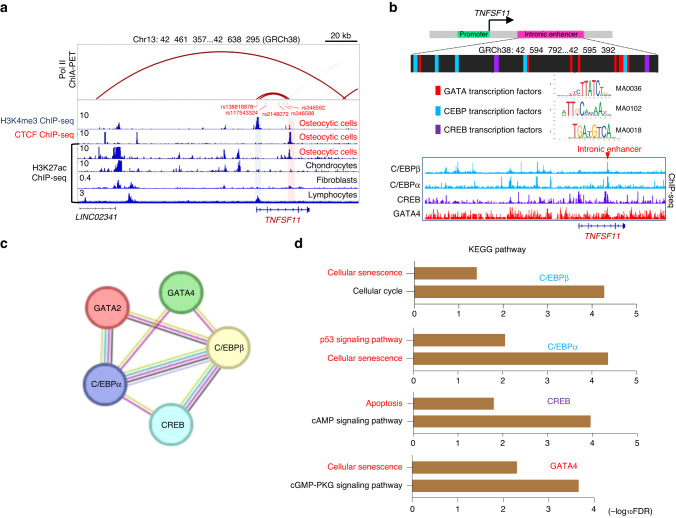
Identification of an intronic enhancer within the *TNFSF11* gene locus in osteocytic cells. **a** H3K4me3, CTCF and H3K27ac ChIP-seq profiles in human osteocytic cells (GSE188004, GSE175159, GSE187956),^22^ chondrocytes (GSE188124),^22^ fibroblasts (GSE128642),^56^ and lymphocytes (ENCFF021TWR) and RNA Pol II ChIA–PET results (ENCFF674MPM) in WTC11 cells within the *TNFSF11* gene locus. The blue and red shaded areas indicate the RANKL promoter region and intronic enhancer region, respectively. Genetic variants associated with bone mineral density (*P* < 5 × 10^–6^) identified in previous studies^25–28^ that are located in the RANKL promoter-intronic enhancer loop are indicated in red letters. **b** Schematic depicting the enriched transcription factor-binding motifs and the predicted binding sites within the intronic enhancer region (upper). ChIP-seq profiles of C/EBPβ (ENCFF679XUJ), C/EBPα (ENCFF988QGQ), CREB1 (ENCFF894ALC) and GATA4 (ENCFF213XUO) (lower). **c**,**d** Protein‒protein interaction network (**c**) and functional enrichment analysis (**d**) of the predicted TFs using the STRING database^58^ (https://string-db.org)

In RANKL-expressing cells such as osteoblasts, synovial fibroblasts and lymphocytes, RANKL expression is tightly controlled by enhancer elements that function in a cell-type-specific manner.^18–21^ To gain insight into the mechanisms underlying RANKL expression in osteocytes, we analyzed publicly available epigenomic datasets of osteocytic cells, chondrocytes, fibroblasts and lymphocytes (GSE188004, GSE175159, GSE187956, GSE188124, GSE128642 and ENCODE project dataset ENCFF021TWR).^22^ Chromatin immunoprecipitation followed by sequencing (ChIP-seq) along with data on antibody binding to acetylated histone H3 Lys27 (H3K27ac) identified an intronic region in the *TNFSF11* gene locus with an enrichment of the active enhancer marker H3K27ac in osteocytic cells but not in other cell types (Fig. 1a). The binding of CCCTC-binding factor (CTCF), which often occurs in active enhancers,^23,24^ was observed in the intronic region in osteocytic cells (Fig. 1a). These data suggest that the intronic region functions as an active enhancer of RANKL in osteocytic cells.

H3K4me3 ChIP-seq analysis using osteocytic cells identified a RANKL promoter region 20 kb upstream of the intronic enhancer, and RNA polymerase II (Pol II) ChIA–PET data (ENCFF674MPM) suggested that there is a chromatin loop between the RANKL promoter and the intronic enhancer (Fig. 1a). Intriguingly, inspection of the genome-wide association study (GWAS) catalog database (https://www.ebi.ac.uk/gwas /) indicated that human polymorphisms associated with bone mineral density (BMD), namely, rs346592,^25^ rs346588,^26^ rs2148072,^27^ rs117543324,^25^ and rs138818878,^26,28^ are located in the promoter-intronic enhancer loop (Fig. 1a), suggesting that these genetic variants may influence bone metabolism by affecting osteocytic RANKL expression in humans.

TF-binding motif analysis for the intronic enhancer region (GRCh38: 42, 594, 792–42, 595, 392) and TF ChIP-seq data analysis suggested the binding of GATA4, C/EBPβ, C/EBPα and CREB to this intronic enhancer (Fig. 1b). The binding sites of these TFs are located in close proximity to one another (Fig. 1b). STRING network analysis predicted potential protein‒protein associations among the TFs (Fig. 1c). Functional enrichment analysis of these TFs revealed enrichment of Kyoto Encyclopedia of Genes and Genomes (KEGG) pathway terms associated with cell senescence and death, such as “cellular senescence”, “p53 signaling pathway” and “apoptosis” (Fig. 1d). These results suggest that signals from cell death and senescence may stimulate the intronic enhancer in osteocytes.

### Dying osteocytic cells highly express RANKL

To test whether cell death signaling stimulates RANKL expression in osteocytes, we analyzed single-cell RNA sequencing (scRNA–seq) data for normal and dying osteocytic cells (GSE154719).^29^ This scRNA–seq dataset consists of tdTomato-positive cells isolated from the long bones of *Dmp1*-Cre; *Sp7*^+/+^;tdTomato (control) and *Dmp1*-Cre;*Sp7*^flox/flox^;tdTomato (*Sp7*^*OcyKO*^) mice.^29^ Osteocyte death was reported to be elevated in the *Sp7*^*OcyKO*^ mice based on terminal deoxynucleotidyl transferase dUTP nick end labeling (TUNEL) staining and immunostaining for activated caspase-3.^29^

**Fig. 2:**
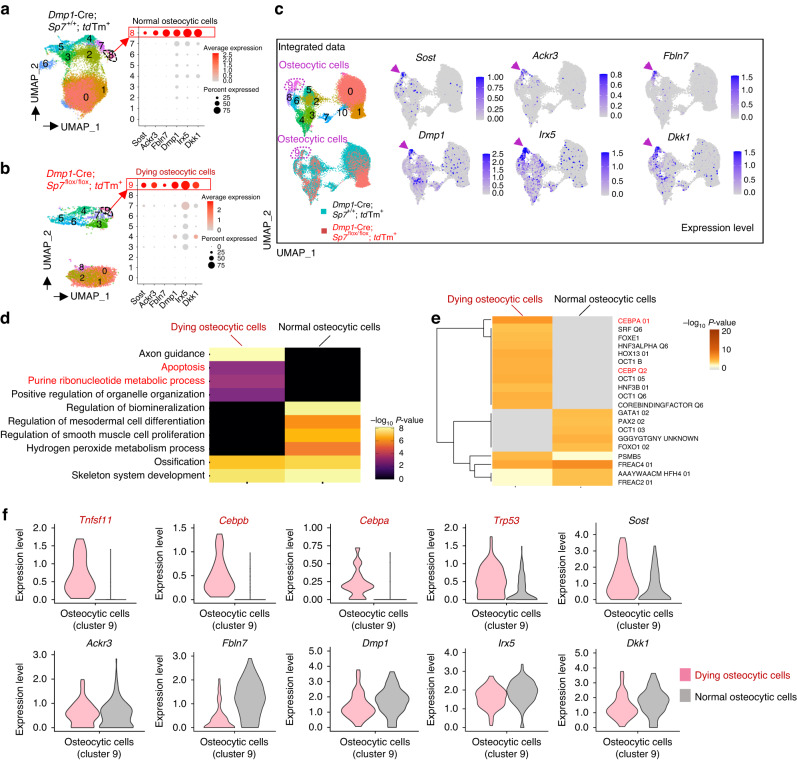
Dying osteocytic cells highly express RANKL. **a**,**b** Uniform manifold approximation and projection (UMAP) plots of the scRNA–seq (GSE154719)^29^ on tdTomato^+^ cells derived from bone tissues of *Dmp1*-Cre;*Sp7*^+/+^;tdTomato^+^ mice (*n* = 21 671 cells) (**a**) and *Dmp1*-Cre;*Sp7*^flox/flox^;tdTomato^+^ mice (*n* = 5 098 cells) (**b**). The dot plots in the right panels show the expression of selected osteocyte marker genes in the identified subclusters. **c** UMAP plot of the integrated data (*n* = 26 769 cells) and feature plots showing the expression of osteocyte marker genes at the single-cell level. The arrowheads indicate Osteocytic Cell Cluster 9. **d**,**e** Functional enrichment analysis (**d**) and regulatory target gene set analysis (**e**) comparing normal osteocytes and dying osteocytic cells. **f** Violin plots showing the expression of selected genes in dying and normal osteocytic cells

As *Dmp1*-Cre is reported to be expressed not only in osteocytes but also in other cells, such as mature osteoblasts,^5^ these scRNA-seq data contained heterogeneous cell populations. Unsupervised clustering analysis identified an osteocytic cell cluster characterized by high expression levels of the osteocyte marker genes *Sost*, *Ackr3, Fbln7, Dmp1*, *Irx5* and *Dkk1* in both control (normal osteocytic cells, Cluster 8) and *Sp7*^*OcyKO*^ mice (dying osteocytic cells, Cluster 9) (Fig. 2a, b). We performed data integration and confirmed that *Sp7* expression was depleted in the osteocytic cells (Cluster 9 in the integrated UMAP) derived from *Sp7*^*OcyKO*^ data (Fig. 2c and Fig. S1A, B), and the Gene Ontology (GO) terms associated with apoptosis were enriched in the *Sp7*-deleted osteocytic cells (Fig. 2d). Motif gene-set enrichment analysis showed that dying osteocytic cells highly expressed genes regulated by factors associated with CEBP, the well-known transcriptional regulator of cellular senescence and apoptosis^30–32^ (Fig. 2e). We found that dying osteocytic cells displayed increased expression levels of *Tnfsf11* as well as the cell senescence/death-related TFs *Cebpb*, *Cebpa* and *Trp53* while maintaining the expression of osteocyte markers such as *Sost*, *Ackr3, Fbln7, Dmp1*, *Irx5* and *Dkk1* (Fig. 2f). There was no difference between normal and dying osteocytic cells in the expression level of osteoprotegerin (OPG, encoded by *Tnfrsf11b*) (Fig. S1C, D). As reported in previous studies,^33,34^ RANKL expression was also detected in bone marrow adipo-progenitor cells (Cluster 4, characterized by high expression levels of *Lpl*, *Kcnk2, Cxcl12* and *Igfbp5*) (Fig. S1A), but *Sp7* deficiency did not affect RANKL expression in adipo-progenitor cells (Fig. S1C, D).

These data suggest that the cell death signal stimulates RANKL expression in osteocytic cells, probably via regulation of the intronic enhancer.

### Generation of intronic enhancer-knockout mice

**Fig. 3:**
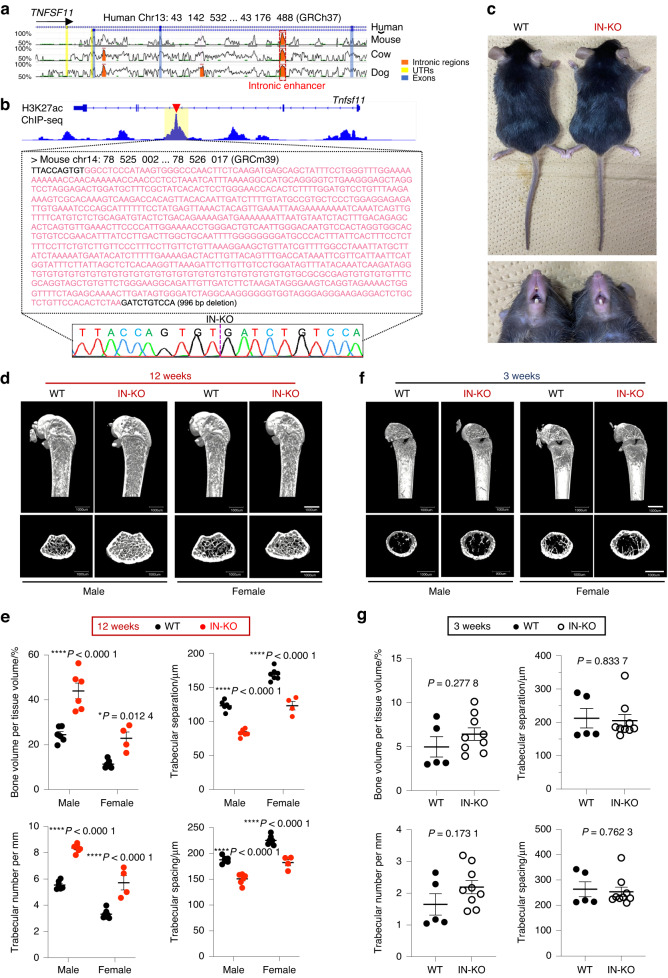
Deletion of the intronic enhancer leads to an age-dependent high-bone-mass phenotype**. a** Sequence similarity analysis of the intronic enhancer among human, mouse, cow and dog genomes using the ECR Browser^59^ (https://ecrbrowser.dcode.org) with 90% ECR similarity across a 300-base-pair ECR length. **b** H3K27ac ChIP-seq profile in murine osteocytic cells (GSE54784).^36^ The nucleic acid sequence of the intronic enhancer in mice is shown in the lower panel. The sequence region colored pink denotes the deletion region obtained with the CRISPR/Cas9 method. **c** Representative macroscopic images of more than three wild-type (WT) and intronic enhancer-knockout (IN-KO) mice (female, 12 weeks). **d**, **e** Representative micro-CT images (**d**) and micro-CT parameters (**e**) of the femur in WT and IN-KO mice at the age of 12 weeks (*n* = 6 male WT and *n* = 6 male IN-KO mice; *n* = 7 female WT and *n* = 4 female IN-KO mice). **f**, **g** Representative micro-CT images (**f**) and micro-CT parameters (**g**) of the femur in WT and IN-KO mice at the age of 3 weeks (*n* = 5 female WT and *n* = 9 female IN-KO mice). Micro-CT scale bars: 1 mm. The data are expressed as the mean ± SEM. *P* values were determined by two-way ANOVA followed by Tukey’s post hoc test (**e**) and two-tailed *t* test (**g**)

Intronic elements often undergo negative selection pressure during the course of evolution, so intron genomic sequences are much less conserved than exons.^23,35^ However, the RANKL intronic enhancer sequence is highly conserved (nearly 90%) among humans, mice, cows and dogs, suggesting that it plays an important role in mammalian homeostasis (Fig. 3a). The murine homologous region of the intronic enhancer also displays enrichment of the H3K27ac binding signal in osteocytic cells (GSE54784),^36^ suggesting a conserved function as an active enhancer of RANKL in osteocytic cells (Fig. 3b). Indeed, this murine homologous region has been shown to display reproducible gene enhancer activity in an in vivo enhancer assay (VISTA Enhancer Browser:^37^ http://enhancer.lbl.gov).

To examine the physiological relevance of this intronic element in vivo, we generated mice lacking the homologous region (GRCm39 chr14: 78, 525, 012–78, 526, 007; 996 bp deletion) of the intronic enhancer using CRISPR–Cas9-mediated genome editing technology. The successful generation of intronic enhancer-KO mice (hereafter called “IN-KO” mice) was confirmed by Sanger DNA sequencing (Fig. 3b). The IN-KO mice were born at the expected Mendelian frequency and displayed normal tooth eruption and a body size similar to that of littermate wild-type controls (Fig. 3c).

Strikingly, IN-KO mice displayed a severe high-bone-mass phenotype at the age of 12 weeks (Fig. 3d). Microcomputed tomography (μCT) analyses showed that IN-KO mice had an increased bone volume per tissue volume and trabecular number along with decreased trabecular separation and trabecular spacing (Fig. 3e). In contrast, there was no difference between the wild-type and IN-KO mice in bone mass at the age of 3 weeks (Fig. 3f, g). This adult stage-restricted high bone mass phenotype of the IN-KO mice is similar to that observed in *Tnfsf11*-floxed mice crossed with *Dmp1*-Cre and *Sost*-Cre mice.^2,3,5^

### Intronic enhancer-deficient mice exhibited decreased osteoclastogenesis and RANKL expression in osteocytic cells

**Fig. 4:**
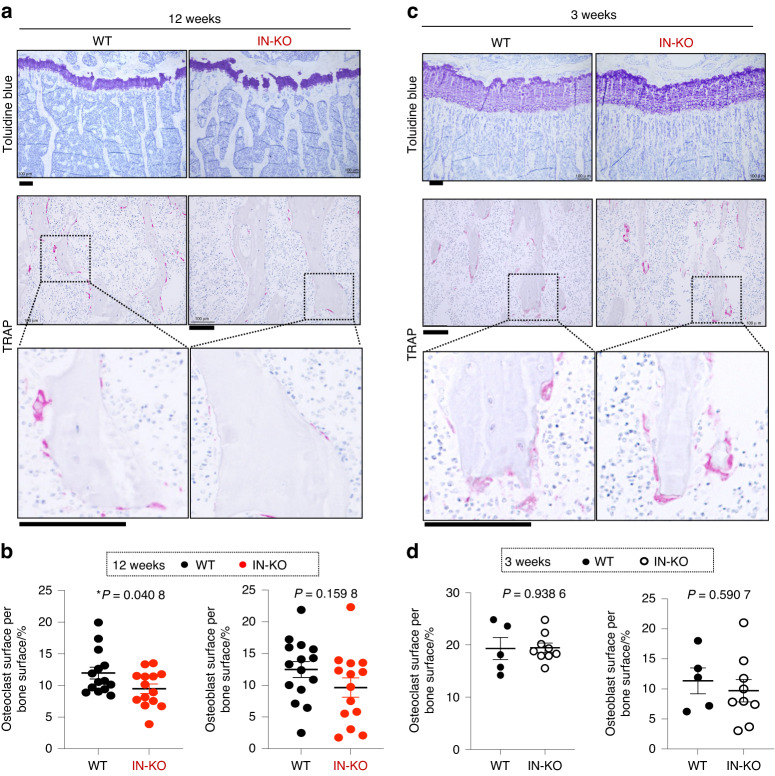
Intronic enhancer deletion inhibits osteoclastogenesis**. a** Toluidine blue and TRAP staining of the proximal tibias of WT and IN-KO mice at the age of 12 weeks. The data are representative of more than 3 independent experiments. **b** Histological analysis of the proximal tibias of WT and IN-KO mice at the age of 12 weeks (WT, *n* = 15; IN-KO, *n* = 14). **c** Toluidine blue and TRAP staining of the proximal tibias of WT and IN-KO mice at the age of 3 weeks. The data are representative of more than 3 independent experiments. **d** Histological analysis of the proximal tibias of WT and IN-KO mice at the age of 3 weeks (WT, *n* = 5; IN-KO, *n* = 9). Histology scale bars: 100 μm. The data are expressed as the mean ± SEM. *P* values were determined by two-tailed *t* test (**b** and **d**)

To understand the cellular basis underlying the high-bone-mass phenotype of IN-KO mice, we performed histological analysis using toluidine blue and tartrate-resistant acid phosphatase (TRAP) staining on bone sections of the wild-type and IN-KO mice. Consistent with the μCT data, toluidine blue staining indicated that the 12-week-old IN-KO mice had a high bone mass phenotype along with an increased trabecular number and thickness (Fig. 4a). Dynamic bone histomorphometry showed that the osteoclast surface per bone surface was significantly decreased in IN-KO mice (Fig. 4b), indicating impaired osteoclastogenesis in these animals. There was no difference between the wild-type and IN-KO mice in osteoblast surface per bone surface (Fig. 4b). At 3 weeks of age, both the osteoclastic and osteoblastic parameters were normal in IN-KO mice (Fig. 4c, d).

**Fig. 5:**
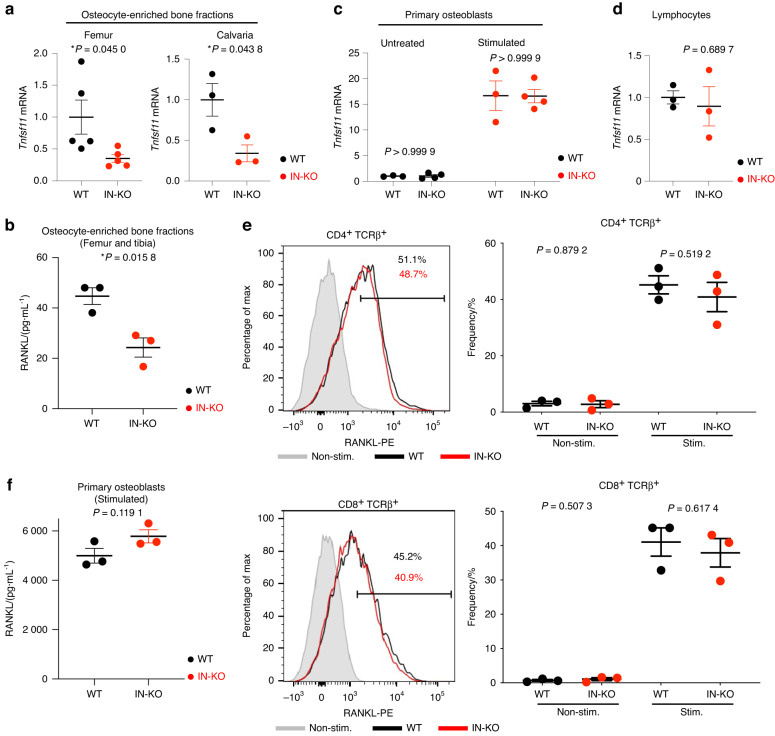
Intronic enhancer deletion decreases RANKL expression in osteocytic cells but not in other cell types. **a** *Tnfsf11* mRNA expression levels in osteocyte-enriched femur (*n* = 5 per group) and calvaria (*n* = 3 per group) bone fractions. **b** RANKL concentration measured by ELISA in the lysate of osteocyte-enriched femurs and tibias (*n* = 3 per group). **c**, **d** *Tnfsf11* mRNA expression levels in primary osteoblasts (*n* = 3 and *n* = 4 in the untreated and vitamin D_3_-treated groups) (**c**) and lymphocytes (*n* = 3 per group) (**d**). **e** Flow cytometric profiles for RANKL expression in CD4 T cells (**e**, upper panel) and CD8 T cells (**e**, lower panel). The T cells were stimulated with plate-bound anti-CD3ε (10 μg·mL^**−**^^1^) for 20 h. The shadow indicates nonstimulated cells. The graphs show the frequency of RANKL-positive T cells. **f** RANKL concentration measured by ELISA in the lysate of calvarial primary osteoblasts stimulated with PGE_2_, 1,25(OH)_2_ D_3_ and TNF-α (*n* = 3 per group). The data are expressed as the mean ± SEM. *P* values were determined by two-tailed *t* test

To test whether RANKL expression was decreased in osteocytes in IN-KO mice, we collected osteocyte-enriched bone fractions by using a previously established method.^5^ The mRNA and protein expression levels of RANKL were significantly decreased in the osteocyte-enriched bone fractions of IN-KO mice (Fig. 5a, b). However, there was no significant difference between the wild-type and IN-KO mice in terms of the RANKL mRNA and protein expression levels in primary osteoblasts or lymphocytes (Fig. 5c–f). These data indicate that the intronic enhancer regulates RANKL expression specifically in osteocytic cells.

Collectively, these data demonstrate that the intronic enhancer controls RANKL expression in osteocytic cells in vivo, thus critically contributing to physiological bone remodeling in the adult stage.

## Discussion

In this study, we identified an intronic enhancer specifically involved in RANKL regulation in osteocytic cells. This intronic enhancer was enriched with binding motifs for GATA, CEBP and CREB TFs, and ChIP-seq data analysis indicated that C/EBPβ, C/EBPα, CREB and GATA4 directly bind to this element. It is well established that C/EBP factors and GATA4 mediate cellular senescence and death.^16,30–32^ CREB is a downstream factor of ATP-induced calcium signaling,^38^ which is suggested to be activated in osteocytes surrounded by dying cells. Since it was first reported by Harold Frost in 1960,^39^ it has become well established that osteocyte death is increased in aged or damaged bones.^4,10–13,15,16,40,41^ As osteocytes are the longest-living cell type in bone tissue, cell death/senescence in osteocytes may reflect places of old or damaged bone that need to be replaced by osteoclasts. We hypothesize that the intronic enhancer may function as a network hub integrating signals from cell death and senescence to induce RANKL expression in osteocytes, telling osteoclasts where to resorb. Since the high-bone-mass phenotype and the reduction in osteoclast number in the IN-KO mice were milder than those in previously reported osteocyte-specific RANKL KO mice,^2,3,5^ it will be important to examine the possibility that other enhancers also contribute to RANKL expression in osteocytes. Further studies are needed to provide a complete picture of the molecular mechanisms underlying RANKL expression in osteocytes.

Consistent with previous reports,^33,34^ RANKL expression was also detected in bone marrow adipo-progenitor cells (Fig. S1A, C, D). The importance of RANKL in adipo-progenitor cells has been proposed by studies using *Tnfsf11*-floxed *Adipoq*-Cre mice.^33,34^ However, the *Adipoq*-Cre system has been shown to target almost all CXCL12-abundant reticular (CAR) cells.^42^ Since CAR cells can give rise to osteoblasts and osteocytes,^43–45^ it is technically difficult to exclude the possibility that the phenotype of *Tnfsf11*-floxed *Adipoq*-Cre mice is influenced by the deletion of RANKL in osteoblasts and osteocytes. Further studies are required to clarify the relative contributions of osteocytes and adipo-progenitor cells as sources of RANKL in bone metabolism and to elucidate the role of the intronic RANKL enhancer in other cell types, including adipo-progenitors.

The importance of osteocyte RANKL in bone remodeling has been demonstrated by studies using *Tnfsf11*-floxed mice crossed with *Dmp1*-Cre and *Sost*-Cre mice.^2,3,5,7,8,11,16^ Although *Dmp1*-Cre can delete targeted gene expression in cell types other than osteocytes under certain conditions, the importance of osteocyte RANKL is supported by the findings of a study using *Sost*-Cre mice, in which Cre recombination occurred only in osteocytes, not in osteoblasts or lining cells.^5^ A previous study using the *Dmp1*-Cre; DTR system showed that diphtheria toxin administration markedly increased RANKL expression in bone tissue.^13^ Other studies have also suggested that osteocyte death activates osteoclastic bone resorption.^46–48^ Given that osteocytes are the major sources of RANKL and the most abundant cells in bone, it has long been enigmatic why osteocyte ablation results in RANKL augmentation and osteoclast activation. Our data suggest that cell death signaling stimulates the intronic enhancer to promote RANKL expression in osteocytes, providing a key missing link that may help explain the conflicting observations in previous reports. Since osteocyte death and RANKL derived from osteocytes have been shown to be involved in bone loss under various pathological conditions, such as osteoporosis, arthritis and osteonecrosis, it will be necessary to examine the pathological significance of the intronic enhancer in future studies.

RANKL is a multifunctional cytokine that plays a central role in the bone and immune systems.^6,20^ RANKL activity is maintained under tight local control, and the main source of RANKL varies among different biological processes.^6^ Previous studies have demonstrated that RANKL expression in osteoblasts, fibroblasts and lymphocytes is regulated by distal enhancers located upstream of the *TNFSF11* gene locus.^18–21,49^ In contrast to other cell types, osteocytic cells utilize regulatory elements located in the intronic region. Intriguingly, the importance of intronic enhancers has also been reported in other genes essential for vertebrate homeostasis, including *Foxp3* in regulatory T cells,^50^ immunoglobulin heavy chain genes in B cells^51^ and *musashi1* in neural stem/progenitor cells.^52^ We suspect that the regulatory element inside the intronic region may exert an advantage in precise control of gene expression by limiting the accessibility of TFs to the open DNA regions and thus avoiding their leaky activity. It is of considerable interest to further investigate why it is necessary for osteocytes to utilize the intronic element to express RANKL and thus ensure skeletal homeostasis.

In summary, bone remodeling is controlled by an osteocytic cell-specific enhancer located in the intronic region of the *TNFSF11* gene (encoding RANKL). This study highlights the cell type-dependent and context-dependent machinery controlling RANKL expression, providing key insights into the regulatory mechanisms underlying vertebrate homeostasis.

## Methods

### Mice

Mice were housed under specific pathogen-free conditions, and all experiments were performed with the approval of the Institutional Review Board at The University of Tokyo. C57BL/6 J (B6) mice were purchased from CLEA Japan. Intronic enhancer-knockout (IN-KO) mice were generated by CRISPR–Cas9-mediated genome editing technology on the C57BL/6 J background. Single-guide (sg) RNA targeting the sequences of the intronic enhancer region (5′-CCTGGACAGATCTTAGAGTG-3′ and 5′-AAGTTGGGCCCACTTATGGG-3′) and *hCas9* mRNA were prepared as described in a previous study.^21^ The primers for the detection of the IN-KO allele were as follows: forward, 5′-TTGGTTGGAAGTGTTGAAATCCC-3′ and reverse, 5′-CAACCAAGTTTTCCCTTGTAGTATCC-3′. The PCR product of the IN-KO allele (175 base pairs) was further sequenced using the forward primer to confirm that the founder progenies harbored intronic region deletion (996 base-pair deletion corresponding to GRCm39 chr14: 78 525 012–78 526 007). Founder progenies were mated with C57BL/6 J WT mice to generate heterozygous offspring and subsequently intercrossed to generate the wild-type and IN-KO littermate progeny. Age- and sex-matched littermates were used for all experiments unless otherwise noted.

### Analysis of the bone phenotype

For microcomputed tomography analysis, femurs were isolated from wild-type and IN-KO mice and fixed with 70% ethanol. Computed tomography scanning was performed using a ScanXmate-A100S Scanner (Comscantechno). Three-dimensional microstructural image data were reconstructed, and structural indices were calculated using TRI/3D-BON software (RATOC). For the histomorphometric analysis, tibias from the wild-type and IN-KO mice were dissected, fixed with 70% ethanol and then subjected to a standard dynamic bone histomorphometric analysis as described previously.^53,54^ Toluidine blue and TRAP staining images were captured using a BZ-II Analyzer (Keyence).

### ChIP-seq, ChIA–PET and GWAS data analyses

ChIP-seq and ChIA–PET data were downloaded from the GEO and ENCODE Project databases. Human osteocytic cell epigenomic datasets (GSE188004 H3K4me3 ChIP-seq, GSE175159 CTCF ChIP-seq and GSE187956 H3K27ac ChIP-seq)^22^ and a chondrocyte epigenomic dataset (GSE188124 H3K27ac ChIP)^22^ were derived from in vitro-differentiated osteocytes and chondrocytes from the stem cell line WA09-WB0143 (WiCell, female, 5 days).^55^ Lymphocyte H3K27ac ChIP-seq ENCFF021TWR data were derived from flow-sorted CD4^+^ primary cells from 1 male (age 37). RNA Pol II ChIA–PET (ENCFF674MPM) was performed on a human induced pluripotent stem cell line (WTC-11). Fibroblast H3K27ac ChIP-seq data (GSE128642)^56^ were derived from rheumatoid arthritis patients who underwent total knee replacement or elbow synovectomy. C/EBPβ ChIP-seq (ENCFF679XUJ) data were derived from mesenchymal stem cells of the bone marrow, and C/EBPα ChIP-seq (ENCFF988QGQ) data were derived from a HepG2 cell line genetically modified (insertion) using CRISPR targeting *H. sapiens* CEBPA. CREB1 ChIP-seq (ENCFF894ALC) was performed on an induced pluripotent stem cell line (GM23338) genetically modified (insertion) using CRISPR targeting *H. sapiens* CREB1. GATA4 ChIP-seq (ENCFF213XUO) was performed on the HepG2 cell line. Murine osteocytic cell H3K27ac ChIP-seq (GSE54784)^36^ was performed on in vitro-differentiated osteocytic cells from the IDG-SW3 osteocyte cell line using osteogenic medium (αMEM culture medium supplemented with 50 μg·mL^**−**1^ ascorbic acid and 4 mmol·L^**−**1^ β-glycerophosphate) for 35 days. We downloaded the GWAS data for bone mineral density (*P* < 5 × 10^–6^) from the GWAS Catalog database (https://www.ebi.ac.uk/gwas), and we mapped it to the RANKL promoter-intronic enhancer loop region and visualized it with the Integrative Genomics Viewer (v2.13.0). We scanned the transcription factor-binding sites with the input sequence of GRCh38 chr13: 42 594 792-42 595 392 using the JASPAR database^57^ (https://jaspar.genereg.net/) and performed protein‒protein interaction network analysis and functional enrichment analysis of the predicted TFs (GATA2, GATA4, C/EBPα, C/EBPβ and CREB) using the STRING database^58^ (https://string-db.org). The ECR Browser^59^ (https://ecrbrowser.dcode.org) with 90% ECR similarity across 300-base-pair ECR lengths was used to analyze the sequence similarity of the intronic enhancer in the human, mouse, cow and dog genomes.

### Computational analyses for scRNA–seq data

The scRNA–seq dataset (GSE154719)^29^ was downloaded from the Gene Expression Omnibus (GEO) database. The samples were prepared from digested bone matrix of Dmp1-Cre;Sp7^+/+^;tdTomato^+^ and Dmp1-Cre;Sp7^flox/flox^;tdTomato^+^ mice, with an aggressive digestion procedure to liberate osteoblasts and matrix-embedded osteocytes (15 min collagenase (0.2% collagenase type I in isolation buffer)/15 min EDTA solution (5 mmol·L^**−**1^ EDTA, 0.1% BSA in PBS)/15 min collagenase solution/15 min EDTA solution/15 min collagenase solution/30 min EDTA solution/30 min collagenase solution/30 min collagenase solution) as described in the original paper.^29^ The viable tdTomato^+^ live cells (collected from the final three collagenase fractions) were sorted and subjected to scRNA–seq. We retrieved the data and performed downstream analysis using the Seurat R package (v4.2.1), including primary analysis, quality control, normalization and scaling, clustering, gene marker identification and visualization of gene expression. Primary filtering removed genes expressed in fewer than 3 cells and cells expressing fewer than 200 genes. Cells with more than 10% mitochondrial reads and 6 000 nFeature_RNA were also removed. After filtering, per-cell counts were normalized (scale factor 10 000 by default), the 2 000 most variable genes using the VST method were identified, and the expression levels of these genes were scaled prior to principal component analysis. The first twenty principal components were used for UMAP projection, and the cells were clustered at a resolution of 1 through a standard graph-based clustering approach. Minor groups of contaminated cells, such as hematopoietic cells (*Ptprc*^+^), perivascular cells (*Ptprc*^–^*Pecam1*^+^) and red blood cells (*Slc4a1*^+^), were removed, and 21 671 cells from *Dmp1*-Cre;*Sp7*^+/+^ samples and 5 098 cells from *Dmp1*-Cre;*Sp7*^flox/flox^ samples were used for further downstream analysis. An osteocytic cell cluster was defined by the expression of well-established osteocyte marker genes such as *Sost, Ackr3, Fbln7, Dmp1, Irx5* and *Dkk1*. We integrated the two datasets using the scRNA–seq integration method described in Seurat^60^ (https://satijalab.org/seurat/articles/integration_introduction.html), resulting in a total of 26 769 cells available for downstream analysis. We used the FindMarkers function to identify genes that were differentially expressed in normal osteocytic cells (derived from *Dmp1*-Cre;*Sp7*^+/+^ samples) and dying osteocytic cells (derived from *Dmp1*-Cre;*Sp7*^flox/flox^ samples). Metascape (https://metascape.org)^61^ was used to perform the enrichment analyses, including the GO biological process, canonical pathway, Reactome gene set, KEGG pathway, WikiPathways, PANTHER pathway and transcription factor target (miscellaneous) analyses. Violin plots were used to visualize the expression of selected genes in normal and dying osteocytic cells.

### ELISA and quantitative RT–PCR analysis

The isolation of the osteocyte-enriched bone fractions was described previously^5^ and is shown in Fig. S2. Briefly, bones were dissected from mice, and soft tissues were removed. In the case of the femur and tibia, the distal and proximal ends were cut off, and bone marrow cells were flushed out using cold PBS. The surface of the bone fraction was then scraped using a scalpel to remove the periosteum, and the bone shaft was cut into small pieces. Bone fractions were then digested using α-MEM solution (Gibco, 11900024) with 0.1% collagenase (Wako Chemicals, 038-22361) and 0.2% Dispase II (Wako Chemicals, 383-02281) a total of 6 times for 15 min each at 37 °C with frequent shaking. After digestion, osteocyte-enriched tissues were collected and subjected to ELISA and qPCR assays. ELISA was performed with a Mouse TRANCE/RANKL/TNFSF11 Quantikine ELISA Kit (R&D SYSTEMS, MTR00). We isolated primary osteoblasts from the calvaria of newborns by enzymatic digestion using α-MEM (Gibco, 11900024) with 0.1% collagenase (Wako Chemicals, 038-22361) and 0.2% Dispase II (Wako Chemicals, 383-02281), as described previously.^21^ The isolated osteoblasts were then incubated in α-MEM containing 10% FBS and 1% antibiotics for 1 day. To examine the RANKL protein levels in osteoblasts by ELISA, calvaria-derived primary osteoblasts were stimulated with 10^–6^ mol·L^**−**1^ prostaglandin E2 (PGE_2_) (Cayman Chemical), 10^–8^ mol·L^**−**1^ 1,25(OH)_2_D_3_ (Wako Chemicals) and 20 ng·mL^**−**1^ tumor necrosis factor-α (TNF-α) (R&D SYSTEMS), as described previously.^54^ Lymphocytes for qPCR assays were prepared from inguinal lymph nodes. Total RNA was extracted from isolated cells using the ReliaPrep RNA Miniprep System (Promega, Z6011) and reverse-transcribed with SuperScript III (Invitrogen, Thermo Fisher Scientific, 11752-250). Quantitative PCR was performed with a LightCycler (Roche) using SYBR Green (Toyobo). The results were normalized to the *Gapdh* expression level. The primers used were *Gapdh*, 5′-AAGGTCATCCCAGAGCTGAA-3′ and 5′-CTGCTTCACCACCTTCTTGA-3′; *Tnfsf11*, 5′-AGCCATTTGCACACCT CAC-3′ and 5′-CGTGGTACCAAGAGGACAGAGT-3′.

### TCR stimulation and flow cytometry

Splenocytes were incubated with biotin-conjugated anti-CD11b (M1/70), anti-CD11c (N418), anti-TER119 (TER-119), anti-NK1.1 (PK136), anti- B220 (RA3-6B2), anti-CD16/32 (93), anti-TCRd (GL3), and anti-Gr1 (RB6-8C5) antibodies on ice for 30 min. Then, splenic T cells were magnetically isolated by using anti-Biotin MicroBeads and LS columns (Miltenyi Biotec). The isolated T cells were washed with and suspended in RPMI 1640 (Fujifilm) supplemented with 10% FCS, 50 μmol·L^**−**1^ 2-mercaptoethanol, 10 mmol·L^**−**1^ HEPES, 2 mmol·L^**−**1^ L-glutamine, 1× nonessential amino acids, 1 mmol·L^**−**1^ sodium pyruvate, 100 U per mL penicillin, and 100 μg·mL^**−**1^ streptomycin. Cells (2.0 × 10^5^) were incubated in 96-well flat bottom plates (non-tissue culture treatment: FALCON) on which anti-CD3ε (145-2C11) was immobilized. After 20 h, stimulated cells were collected and stained with anti-CD4 (GK1.5), anti-CD8α (5H10–1), anti-TCRβ (H57–597), and anti-RANKL (IK22/5) at final concentrations of 1–2 μg·mL^**−**1^. Flow cytometric analysis was performed with a FACSCanto II (BD Biosciences). 7-Aminoactinomycin D (Fujifilm) was used to exclude dead cells. All antibodies were purchased from BioLegend.

### Statistics

The data were analyzed using GraphPad Prism software v9.4.1 and R software v4.2.0. The statistical tests, *n* values, replicate experiments and *P* values are all indicated in the figures and/or legends. *P* values were calculated using Student’s *t* test and ANOVA with Tukey’s post hoc test.

### Supplementary information

Supplementary Figure 1

Supplementary Figure 2

Supplementary Figures Legend

## Data Availability

All the data that support the plots within this paper are available in the main text. The referenced publicly available scRNA–seq (GSE154719) and ChIP-seq data (GSE188004, GSE175159, GSE187956, GSE54784, GSE188124 and GSE128642) were downloaded from the GEO database. The ChIP-seq datasets ENCFF021TWR, ENCFF679XUJ, ENCFF894ALC, ENCFF988QGQ, and ENCFF213XUO and the RNA Pol II ChIA–PET dataset ENCFF674MPM were downloaded from the ENCODE Project database. The R scripts for data analysis used in this study are available at GitHub.

## References

[1] Bonewald LF (2011). The amazing osteocyte. J. Bone Min. Res..

[2] Nakashima T (2011). Evidence for osteocyte regulation of bone homeostasis through RANKL expression. Nat. Med..

[3] Xiong J (2011). Matrix-embedded cells control osteoclast formation. Nat. Med..

[4] Xiong J, O'Brien CA (2012). Osteocyte RANKL: new insights into the control of bone remodeling. J. Bone Min. Res..

[5] Xiong J (2015). Osteocytes, not osteoblasts or lining cells, are the main source of the RANKL required for osteoclast formation in remodeling bone. PLoS One.

[6] Tsukasaki M, Takayanagi H (2019). Osteoimmunology: evolving concepts in bone-immune interactions in health and disease. Nat. Rev. Immunol..

[7] Fujiwara Y (2016). RANKL (Receptor Activator of NFκB Ligand) produced by osteocytes is required for the increase in B cells and bone loss caused by estrogen deficiency in mice. J. Biol. Chem..

[8] Xiong J (2014). Osteocyte-derived RANKL is a critical mediator of the increased bone resorption caused by dietary calcium deficiency. Bone.

[9] Wein MN (2018). Parathyroid hormone signaling in osteocytes. J. Bone Min. Res.

[10] O'Brien CA (2004). Glucocorticoids act directly on osteoblasts and osteocytes to induce their apoptosis and reduce bone formation and strength. Endocrinology.

[11] Piemontese M, Xiong J, Fujiwara Y, Thostenson JD, O'Brien CA (2016). Cortical bone loss caused by glucocorticoid excess requires RANKL production by osteocytes and is associated with reduced OPG expression in mice. Am. J. Physiol. Endocrinol. Metab..

[12] Weinstein RS (2011). Osteoprotegerin prevents glucocorticoid-induced osteocyte apoptosis in mice. Endocrinology.

[13] Tatsumi S (2007). Targeted ablation of osteocytes induces osteoporosis with defective mechanotransduction. Cell Metab..

[14] Cheung WY (2016). Pannexin-1 and P2X7-Receptor are required for apoptotic osteocytes in fatigued bone to trigger RANKL production in neighboring bystander osteocytes. J. Bone Min. Res..

[15] Andreev D (2020). Osteocyte necrosis triggers osteoclast-mediated bone loss through macrophage-inducible C-type lectin. J. Clin. Investig..

[16] Kim HN (2020). Osteocyte RANKL is required for cortical bone loss with age and is induced by senescence. JCI Insight.

[17] Jilka RL (2014). Dysapoptosis of osteoblasts and osteocytes increases cancellous bone formation but exaggerates cortical porosity with age. J. Bone Min. Res..

[18] Onal M (2016). Unique distal enhancers linked to the mouse Tnfsf11 gene direct tissue-specific and inflammation-induced expression of RANKL. Endocrinology.

[19] Fu Q, Manolagas SC, O'Brien CA (2006). Parathyroid hormone controls receptor activator of NF-kappaB ligand gene expression via a distant transcriptional enhancer. Mol. Cell Biol..

[20] O'Brien CA (2010). Control of RANKL gene expression. Bone.

[21] Yan M (2022). ETS1 governs pathological tissue-remodeling programs in disease-associated fibroblasts. Nat. Immunol..

[22] ENCODE Project Consortium. An integrated encyclopedia of DNA elements in the human genome. *Nature* **489**, 57–74 (2012). 10.1038/nature11247 PMC3439153 22955616

[23] Shen Y (2012). A map of the cis-regulatory sequences in the mouse genome. Nature.

[24] Holwerda SJ, de Laat W (2013). CTCF: the protein, the binding partners, the binding sites and their chromatin loops. Philos. Trans. R. Soc. Lond. B Biol. Sci..

[25] Kim SK (2018). Identification of 613 new loci associated with heel bone mineral density and a polygenic risk score for bone mineral density, osteoporosis and fracture. PLoS One.

[26] Morris JA (2019). An atlas of genetic influences on osteoporosis in humans and mice. Nat. Genet..

[27] Kemp JP (2014). Phenotypic dissection of bone mineral density reveals skeletal site specificity and facilitates the identification of novel loci in the genetic regulation of bone mass attainment. PLoS Genet..

[28] Kichaev G (2019). Leveraging Polygenic Functional Enrichment to Improve GWAS Power. Am. J. Hum. Genet..

[29] Wang JS (2021). Control of osteocyte dendrite formation by Sp7 and its target gene osteocrin. Nat. Commun..

[30] Kuilman T (2008). Oncogene-induced senescence relayed by an interleukin-dependent inflammatory network. Cell.

[31] Acosta JC (2008). Chemokine signaling via the CXCR2 receptor reinforces senescence. Cell.

[32] Kang C (2015). The DNA damage response induces inflammation and senescence by inhibiting autophagy of GATA4. Science.

[33] Hu Y (2021). RANKL from bone marrow adipose lineage cells promotes osteoclast formation and bone loss. EMBO Rep..

[34] Yu W (2021). Bone marrow adipogenic lineage precursors promote osteoclastogenesis in bone remodeling and pathologic bone loss. J. Clin. Investig.

[35] Levine M (2010). Transcriptional enhancers in animal development and evolution. Curr. Biol..

[36] St John HC (2014). The osteoblast to osteocyte transition: epigenetic changes and response to the vitamin D3 hormone. Mol. Endocrinol..

[37] Visel A, Minovitsky S, Dubchak I, Pennacchio LA (2007). VISTA Enhancer Browser–a database of tissue-specific human enhancers. Nucleic Acids Res..

[38] Wen AY, Sakamoto KM, Miller LS (2010). The role of the transcription factor CREB in immune function. J. Immunol..

[39] Frost HM (1960). In vivo osteocyte death. J. Bone Jt. Surg. Am..

[40] Plotkin LI (2014). Apoptotic osteocytes and the control of targeted bone resorption. Curr. Osteoporos. Rep..

[41] McKenzie J (2019). Osteocyte Death and Bone Overgrowth in Mice Lacking Fibroblast Growth Factor Receptors 1 and 2 in Mature Osteoblasts and Osteocytes. J. Bone Min. Res..

[42] Mukohira H (2019). Mesenchymal stromal cells in bone marrow express adiponectin and are efficiently targeted by an adiponectin promoter-driven Cre transgene. Int. Immunol..

[43] Omatsu Y (2010). The essential functions of adipo-osteogenic progenitors as the hematopoietic stem and progenitor cell niche. Immunity.

[44] Zhou BO, Yue R, Murphy MM, Peyer JG, Morrison SJ (2014). Leptin-receptor-expressing mesenchymal stromal cells represent the main source of bone formed by adult bone marrow. Cell Stem Cell.

[45] Seike M, Omatsu Y, Watanabe H, Kondoh G, Nagasawa T (2018). Stem cell niche-specific Ebf3 maintains the bone marrow cavity. Genes Dev..

[46] Verborgt O, Gibson GJ, Schaffler MB (2000). Loss of osteocyte integrity in association with microdamage and bone remodeling after fatigue in vivo. J. Bone Min. Res..

[47] Jilka RL, Noble B, Weinstein RS (2013). Osteocyte apoptosis. Bone.

[48] Noble BS (2003). Mechanical loading: biphasic osteocyte survival and targeting of osteoclasts for bone destruction in rat cortical bone. Am. J. Physiol. Cell Physiol..

[49] Bishop KA (2015). Transcriptional regulation of the human TNFSF11 gene in T cells via a cell type-selective set of distal enhancers. J. Cell Biochem..

[50] Zheng Y (2010). Role of conserved non-coding DNA elements in the Foxp3 gene in regulatory T-cell fate. Nature.

[51] Gillies SD, Morrison SL, Oi VT, Tonegawa S (1983). A tissue-specific transcription enhancer element is located in the major intron of a rearranged immunoglobulin heavy chain gene. Cell.

[52] Kawase S (2011). Identification of a novel intronic enhancer responsible for the transcriptional regulation of musashi1 in neural stem/progenitor cells. Mol. Brain.

[53] Tsukasaki M (2022). Periosteal stem cells control growth plate stem cells during postnatal skeletal growth. Nat. Commun..

[54] Asano T (2019). Soluble RANKL is physiologically dispensable but accelerates tumour metastasis to bone. Nat. Metab..

[55] Menendez L, Yatskievych TA, Antin PB, Dalton S (2011). Wnt signaling and a Smad pathway blockade direct the differentiation of human pluripotent stem cells to multipotent neural crest cells. Proc. Natl. Acad. Sci. USA.

[56] Loh C (2019). TNF-induced inflammatory genes escape repression in fibroblast-like synoviocytes: transcriptomic and epigenomic analysis. Ann. Rheum. Dis..

[57] Castro-Mondragon JA (2022). JASPAR 2022: the 9th release of the open-access database of transcription factor binding profiles. Nucleic Acids Res..

[58] Szklarczyk D (2021). The STRING database in 2021: customizable protein-protein networks, and functional characterization of user-uploaded gene/measurement sets. Nucleic Acids Res..

[59] Ovcharenko I, Nobrega MA, Loots GG, Stubbs L (2004). ECR Browser: a tool for visualizing and accessing data from comparisons of multiple vertebrate genomes. Nucleic Acids Res..

[60] Hao Y (2021). Integrated analysis of multimodal single-cell data. Cell.

[61] Zhou Y (2019). Metascape provides a biologist-oriented resource for the analysis of systems-level datasets. Nat. Commun..

