# Supplementary figures and images for: Identification of an intronic enhancer regulating RANKL expression in osteocytic cells
Source: Bone Res. 2023 Aug 11;11:43. doi: 10.1038/s41413-023-00277-6 (PMC10415388; doi:10.1038/s41413-023-00277-6)

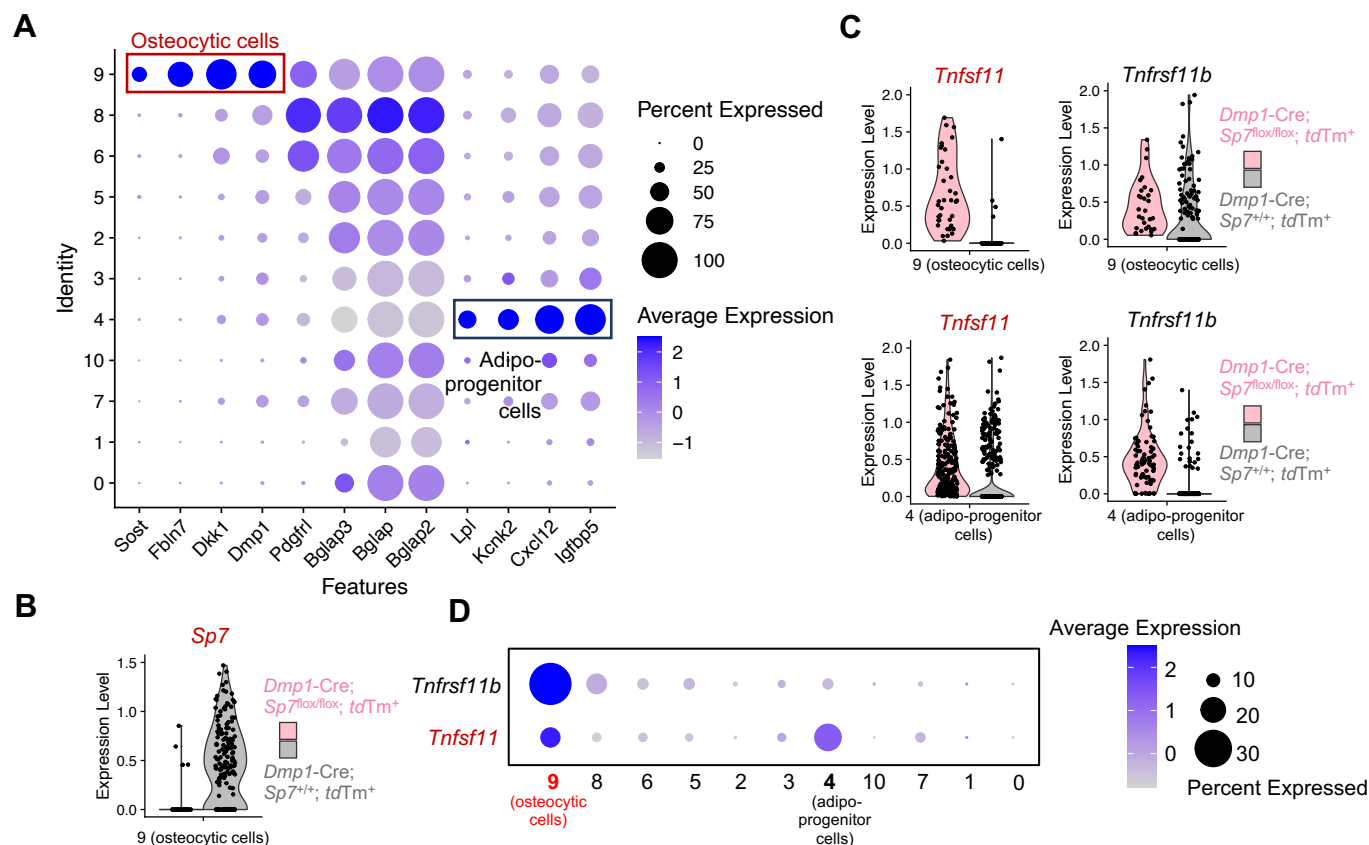

Supplement: Supplementary file 1 — Supplementary Figure 1 [file 41413_2023_277_MOESM1_ESM.pdf]
