# Supplementary material for: Identification of an intronic enhancer regulating RANKL expression in osteocytic cells
Source: Bone Res. 2023 Aug 11;11:43. doi: 10.1038/s41413-023-00277-6 (PMC10415388; doi:10.1038/s41413-023-00277-6)
Supplement: Supplementary file 2 — Supplementary Figure 2 [file 41413_2023_277_MOESM2_ESM.pdf]

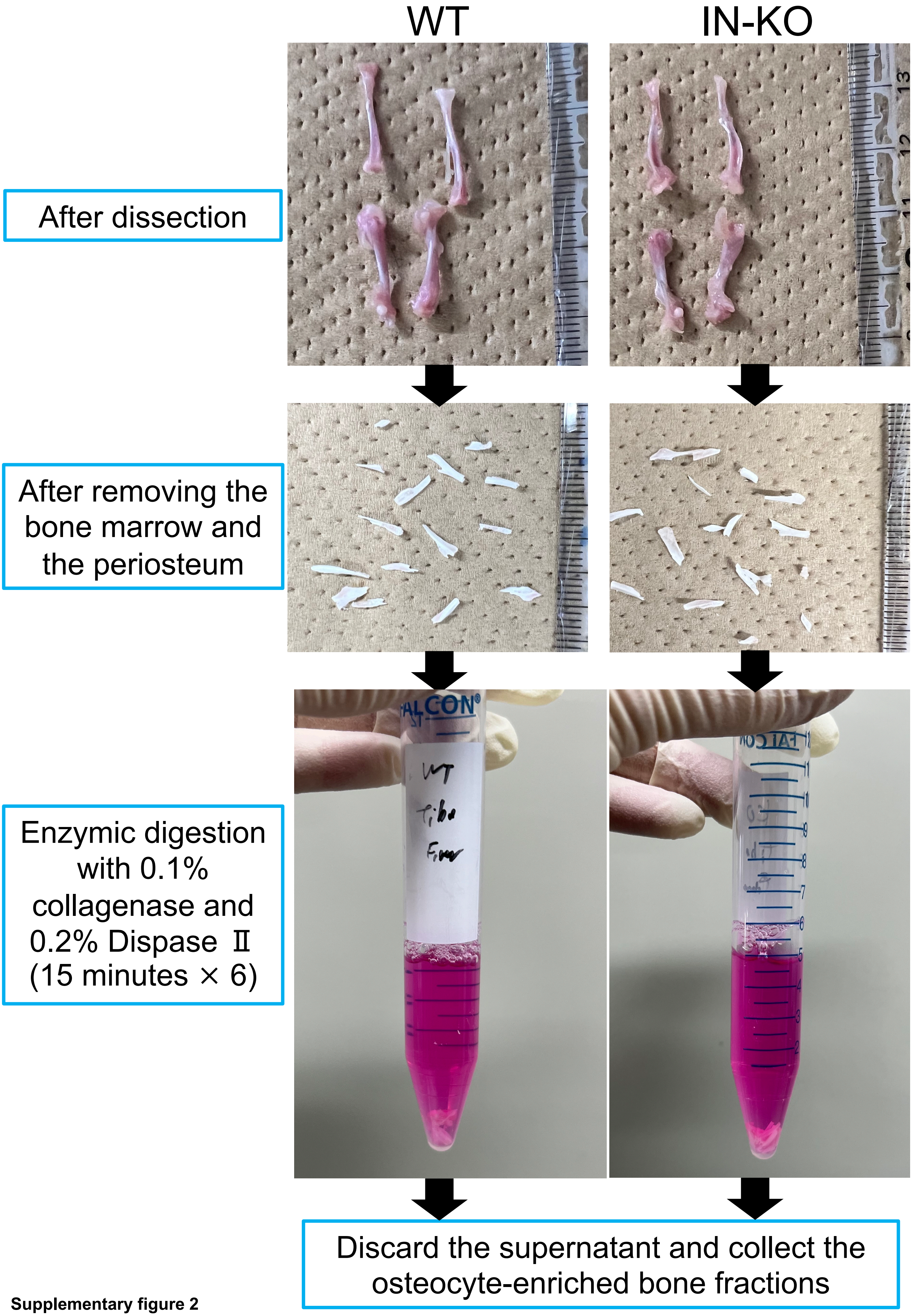

Supplementary figure 2
