# Supplementary material for: Identification of an intronic enhancer regulating RANKL expression in osteocytic cells
Source: Bone Res. 2023 Aug 11;11:43. doi: 10.1038/s41413-023-00277-6 (PMC10415388; doi:10.1038/s41413-023-00277-6)
Supplement: Supplementary file 3 — Supplementary Figures Legend [file 41413_2023_277_MOESM3_ESM.docx]

**Supplementary Figure Legends**

**Supplementary Figure 1. RANKL and OPG expression levels in osteocytic cells and adipo-progenitor cells.**

**(A)** Dot plot showing the expression of selected marker genes in the identified subclusters corresponding to the integrated data shown in Figure 2C. (**B**) Violin plot showing the expression of *Sp7* in osteocytic cells (Cluster 9) of *Dmp1*-Cre;*Sp7*^+/+^;tdTomato^+^ and *Dmp1*-Cre;*Sp7*^flox/flox^;tdTomato^+^ datasets. (**C and D**) Violin plots (C) and dot plot (D) showing the expression of *Tnfsf11* (encoding RANKL) and *Tnfrsf11b* (encoding OPG) in osteocytic cells (Cluster 9) and adipo-progenitor cells (Cluster 4).

**Supplementary Figure 2. Method for isolation of osteocyte-enriched bone tissues**

The distal and proximal ends of the femur and tibia were cut off, and bone marrow cells were flushed out using cold PBS. The surface of the bone fraction was then scraped using a scalpel to remove the periosteum, and the bone shaft was cut into small pieces. The bone fractions were then digested with 0.1% collagenase and 0.2% Dispase II for a total of 6 times with 15 minutes for each at 37 °C with frequent shaking. After digestion, osteocyte-enriched tissues were collected and subjected to ELISA and qPCR assays.
